# Pancreatic Carcinoma Metastatic to the Gingiva

**DOI:** 10.3390/clinpract11010010

**Published:** 2021-01-31

**Authors:** Arijan Zubović, Margita Belušić-Gobić, David Harmicar, Jasna Marušić, Damir Vučinić, Gordana Zamolo

**Affiliations:** 1 Clinic for Maxillofacial Surgery, Clinical Hospital Center Rijeka, 51000 Rijeka, Croatia; arijan.zubovic@gmail.com (A.Z.); margita.belusic@gmail.com (M.B.-G.); david.harmicar@uniri.hr (D.H.); 2 Clinic of Radiotherapy and Oncology, Clinical Hospital Center Rijeka, 51000 Rijeka, Croatia; jasnaskrobonja@yahoo.com; 3 Department of Pathology, Faculty of Medicine, University of Rijeka, 51000 Rijeka, Croatia; gordanazamolo@yahoo.com

**Keywords:** adenocarcinoma, metastasis, pancreatic cancer, immunohistochemistry

## Abstract

Metastatic tumors to the oral cavity are uncommon, representing approximately 1% of all cases of oral malignant lesions even when a metastatic disease is present. The 53-year-old female is presented complaining of abdominal pain, weight loss, and a loose stool recurring not more than three times per day. A computed tomography (CT) scan of the abdomen showed a retroperitoneal mass expanding along the body of the pancreas. Colonoscopy and gastroscopy with a gastric mucosa biopsy showed a normal result. After laparoscopic surgery, the primary site of adenocarcinoma was not confirmed. The patient was referred to the Maxillofacial Surgery Clinic with pain, swelling, and occasional bleeding around the lower right second mollar. Immunohistochemicaly, the tumor cells were positive for Cytokeratin (CK) 19, Cytokeratin (CK) 7, and homebox protein (CDX-2), which are highly sensitive markers of pancreatobiliar cancer. Therefore, the patient was diagnosed with pancreatic carcinoma. This report describes a rare metastasis of malignant pancreatic tumor to the lower right gingiva and highlights the importance of immunohistochemical examination and how it helped identify both the origin and the nature of gingival neoplasm.

## 1. Introduction

Metastatic tumors to the oral cavity are uncommon, representing approximately 1% of all cases of oral malignant lesions [1]. Gnathic bones were affected more commonly than the soft tissues of the oral cavity [2,3]. The prevalence of these tumors in the tongue is reported to be 0.2% [2]. Patients with pancreatic cancer rarely have symptoms in the early stage, and common symptoms that are present are usually non-specific, including shoulder and back pain, dysphagia, dyspepsia, lethargy, and changes in bowel habit [4,5]. In accordance with that, patients with pancreatic carcinoma at the initial diagnosis have metastatic disease [5]. Sometimes, the intraoral metastasis may be the first sign of an underlying cancer in the body [6]. In this case, biopsy of the metastatic lesion, histology, and immunochemistry played an important role for the primary tumor differentiation. This report describes a rare metastasis of malignant pancreatic tumor to the lower right gingiva and highlights the importance of an accurate diagnosis and treatment when presenting symptoms and signs are inconclusive.

## 2. Case Report

**Figure 1:**
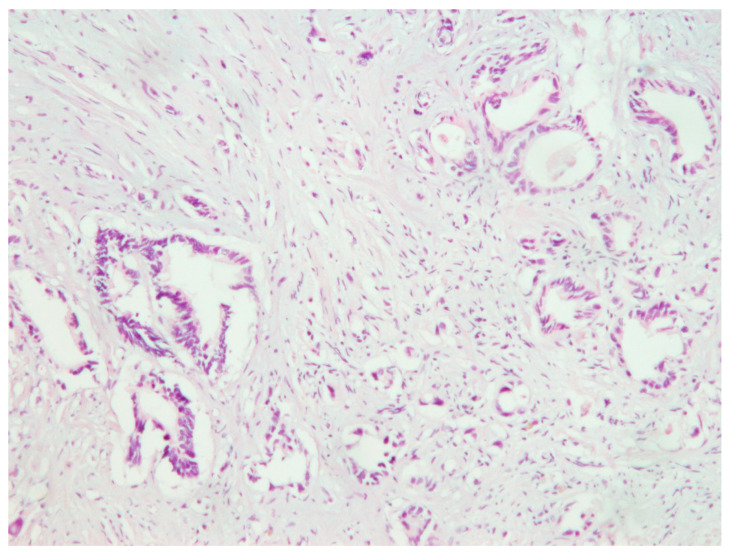
Retroperitoneal tumor mass biopsy suggested gastrointestinal adenocarcinoma, H&E (100×).

The 53-year-old female presented complaining with abdominal pain, weight loss, and loose stool recurring not more than three times per day. The patient had been healthy, with no history of chronic illness. Ultrasound of the abdomen in 2017 showed a hyperechoic pancreas with hypodensity of the pancreatic body. Laboratory data showed normal CA-19-9 serum levels at 32.9 (reference values 0.0–35.0 U/mL). Computed tomography (CT) scan of the abdomen showed a large calcified retroperitoneal mass medially to the left adrenal gland. For the purpose of taking a tissue specimen for pathohistological diagnostics, explorative laparotomy was done. The large tumor mass infiltrated the abdominal aorta, inferior vena cava, truncus celiacus, and the body of the pancreas. The biopsy confirmed adenocarcinoma, primarily of the colon (Figure 1). After surgery, laboratory data showed an increased level of CA-19-9 at 51.8.

**Figure 2:**
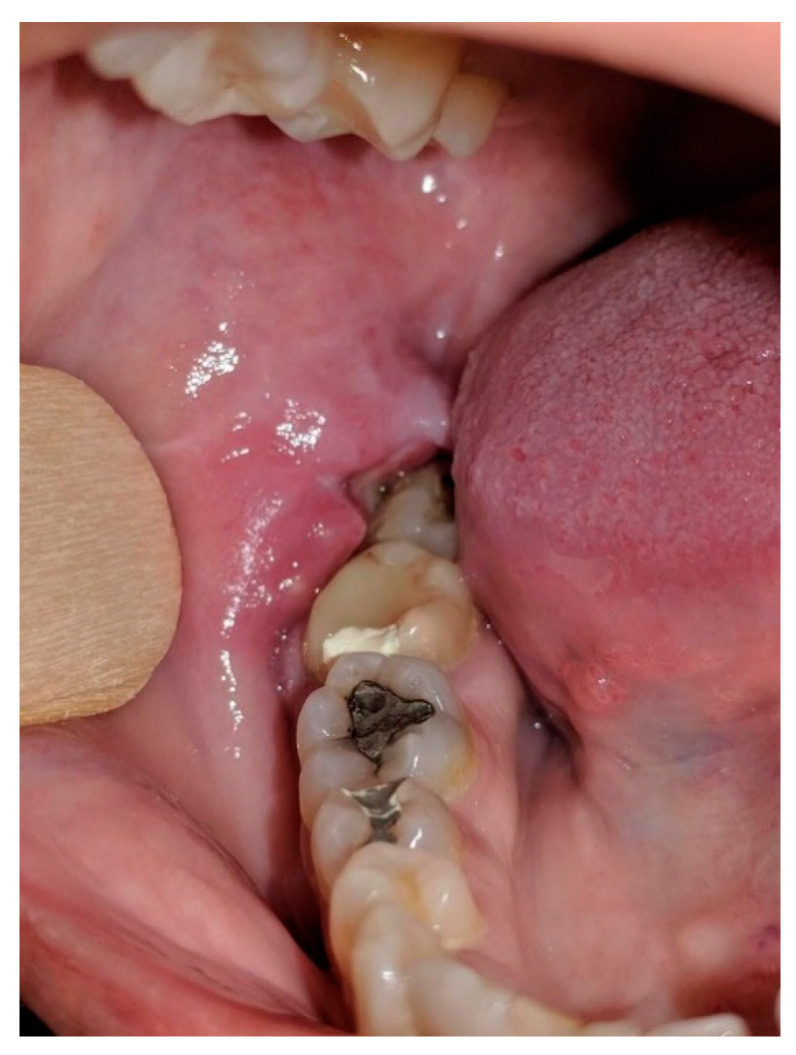
Gingival metastasis of pancreatic adenocarcinoma, 2 × 1.5 cm nodule, unclear margin with soft texture and partial mucosal ulceration.

**Figure 3:**
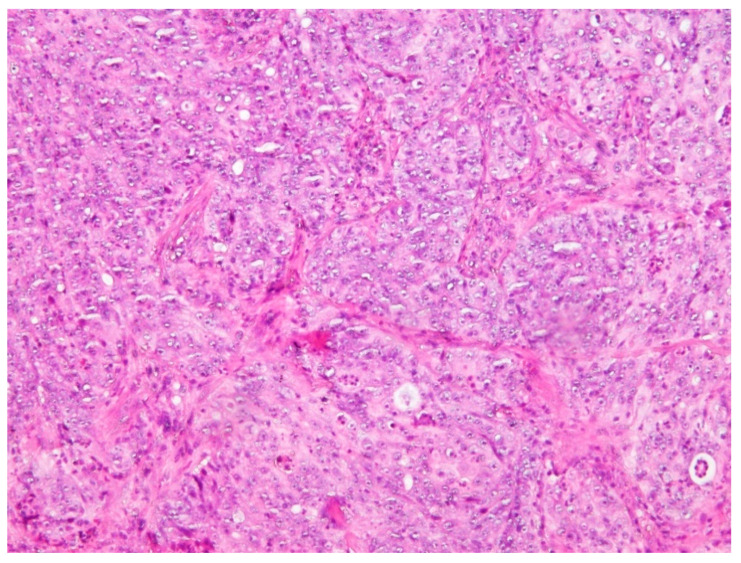
Solid nests of atypical epithelial cells with nuclear polymorphism and mitotic activity separated by connective tissue (H&E 200×).

**Figure 4:**
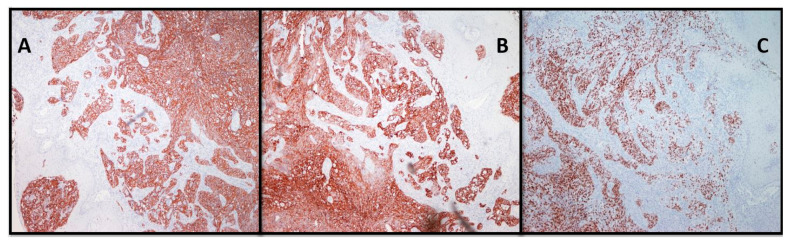
Strong immunoreactivity of the tumor cells for Cytokeratin (CK) 19 (**A**), Cytokeratin (CK) 7 (**B**) and homebox protein (CDX-2) (**C**) (100×).

Postoperative PET/CT scan of the abdomen showed a retroperitoneal mass expanding along the body of the pancreas, which included the left adrenal gland and truncus celiacus. Continuing, the scan showed a soft tissue mass in right upper lobe of the lungs. Both masses showed an increased uptake of the FDG. Colonoscopy and gastroscopy with a gastric mucosa biopsy showed a normal result. Revision of the biopsy was performed after laparotomy was done, and it was immunohistochemically confirmed that the primary lesion is originating either from the colon or pancreas. The patient started treatment with a total of 10 cycles of FOLFOX (FOL—Folinic acid, F—Fluorouracil, OX—Oxaliplatin) chemotherapy regimen and received the last dose in May of 2018. After the 6th cycle of FOLFOX regimen therapy, PET/CT scan showed an expansive mass retroperitoneally with mild morphological and metabolical regression but with a still active malignant disease. A nodal mass in the upper right lobe of the lungs also showed metabolic and morphological regression. However, tumor marker CA 19-9 serum level was elevated to 350.6 U/mL in August 2018. In that time, the patient complained of abdominal pain, weight loss, loss of appetite, and pain in the right part of the oral cavity. She was referred in September of 2018 to the Maxillofacial Surgery Clinic for further examination of the lesion and for an incision biopsy. She presented with pain, swelling, and occasional bleeding around the lower right second molar. Physical examination showed a 2 × 1.5 cm nodule, an unclear margin with soft texture, and a partial mucosal ulceration (Figure 2). There was no bony destruction from radiography. Under local infiltration anesthesia, biopsy of the lesion was done. It was reported as a poorly differentiated carcinoma. The surface of the lesion had a hyperkeratotic epithelium, while the dermis tumor tissue was built of solid clusters of atypical epithelial cells, in part vesicular nuclei and prominent nucleoli, forming glandular formations. Immunohistochemically, the tumor cells were positive for Cytokeratin (CK) 19, Cytokeratin (CK), 7 and homebox protein (CDX-2), which are highly sensitive markers of pancreatobiliar cancer (Figure 3 and Figure 4). Therefore, the patient was diagnosed with pancreatic carcinoma, based on a CT scan of the abdomen, endoscopic ultrasound, biopsy of the gingival lesion, histopathological examination, and immunohistochemistry. After the final diagnosis, the patient started gemcitabine and nab–paclitaxel combination chemotherapy treatment. Unfortunately, the patient did not respond to chemotherapy and she died from the disease a year and a half after her initial diagnosis.

## 3. Discussion

To best of our knowledge this is a rare report of a metastasis of pancreatic adenocarcinoma to the mandibular gingiva. In this case, oral metastasis was not the first indication of pancreatic carcinoma, but histological examination helped confirm the diagnosis. Metastatic tumors within the oral cavity are very uncommon. The head and neck region is usually not included in the staging scans for abdominal malignancies, so some micrometastases may pass undetected. Therefore, the incidence of metastatic diseases that affect the oral cavity is still unknown. Signs and symptoms which are commonly reported, as in our case, include pain in the affected area, bleeding, swelling, and discomfort [7,8]. Metastatic gingival malignancy presents usually as a soft hyperemic nodule with or without pain [9,10]. In Table 1, we provide a short literature review of case reports presenting patients with pancreatic cancer metastases to the oral cavity.

Pancreatic neoplasm is an aggressive malignancy with 20–30% of potentially curable cases, which are localized when diagnosed. The overall 5-year survival rate of pancreatic neoplasms is approximately 5% [11]. Metastases of regional lymph nodes are more common than metastases of distant organs. Hematogenous spreading is usually found in liver (64% to 80%), peritoneum (40% to 55%), and lungs (27% to 50%) [12]. Carcinomas that metastasize more commonly to the oral cavity include prostate, breast, kidney, lung, and gastrointestinal malignancies [13,14,15]. Oral metastasis usually occurs between the fourth and seventh decade of life, where the mandible is more commonly affected than the maxilla [13,14]. Pathogenesis of the gingival metastasis is probably associated with an oral inflammation that possibly attracts migration and adhesion of the cancer cells to the gingiva. Chronic inflammation has been involved in tumorigenesis, including cellular transformation, proliferation, survival, invasion, and metastasis. The presence of some inflammatory molecules and the rich capillary network of the inflamed gingiva may favor the further progression of metastatic cells. Future investigation of this type of mechanism remains to be elucidated [16]. Metastatic disease in the oral cavity from pancreatic adenocarcinoma can be a cause of confusion pathohistologically because of the rarity of such lesions. Therefore, a timely biopsy is suggested in order to exclude a metastatic oral cancer, which can be easily confused with benign reactive lesions or inflammation [9]. Immunohistochemical examination helped to identify both the origin and the nature of gingival neoplasm. Molecular biomarkers are also the subject of discussion in primary oral tumors. The great heterogeneity among these tumors is the reason for why we still do not have suitable biomarkers for their diagnosis. In metastatic tumors of the oral cavity, this heterogeneity is even greater [17]. Excision of the metastasis might help with the patient survival, although it is palliative in most cases [18,19].

The diagnosis of a metastasis in the oral cavity poses a considerable challenge and the practitioner needs to be careful of the risk these lesions present, while the pathologist has to determine the original tumor based on immunohistochemistry. This helps to determine and to increase the chances of a successful differential diagnosis. Various technologies such as the VELscope fluorescence method are used today to help diagnose primary and metastatic lesions of the oral cavity [20,21]. Metastatic lesions should always be included in the differential diagnosis, so the treatment can be started more promptly. Management strategies in cases such as this should be approached by a multidisciplinary team. Such an approach represents the key for success of the primary oral cancer treatment but even more so for metastatic lesions.

## Figures and Tables

**Table 1:** Review of case reports presenting patients with metastases of pancreatic cancer in the oral cavity.

Authors	Year Reported	Patient Age	Patient Sex	Smoking	Previous Diseases of the Oral Cavity	Clinical Presentation	Site of Metastasis	PHD
Stechera et al. [9]	1981	46	Male	Yes	Chronic periodontitis	Teeth 21–27 were extremely mobile, attached to inflammed, edematous gingival tissue	Mandibular gingiva	Adenocarcinoma
Kucuktulu et al. [10]	2013	72	Male	Unknown	No	PET/CT evaluation revealed a 2 × 1 mm mass, hard in consistency in right anterior 1/3 of the tongue	Tongue	Adenocarcinoma
Maschino et al. [19]	2013	71	Male	Yes	Chronic periodontitis	Difficulties healing after oral surgery	Maxillar gingiva	Adenocarcinoma
Kim et al. [3]	2005	Unknown	Unknown	Unknown	Unknown	bleeding and increasing tooth mobility.	Unknown	Pleomorphic carcinoma

## Data Availability

No new data were created or analyzed in this study. Data sharing is not applicable to this article.
